# Maternal Metabolites Indicative of Mental Health Status during Pregnancy

**DOI:** 10.3390/metabo13010024

**Published:** 2022-12-23

**Authors:** Katarina Laketic, Sophie Lalonde-Bester, Kim Smyth, Donna M. Slater, Suzanne C. Tough, Hiroaki Ishida, Hans J. Vogel, Gerald F. Giesbrecht, Chunlong Mu, Jane Shearer

**Affiliations:** 1 Alberta Children’s Hospital Research Institute, Cumming School of Medicine, University of Calgary, Calgary, AB T2N 4N1, Canada; 2 Department of Pediatrics and Community Health Sciences, Cumming School of Medicine, University of Calgary, Calgary, AB T2N 4N1, Canada; 3 Department of Physiology and Pharmacology, Cumming School of Medicine, University of Calgary, Calgary, AB T2N 4N1, Canada; 4 Department of Biological Sciences, Faculty of Science, University of Calgary, Calgary, AB T2N 1N4, Canada; 5 Department of Biochemistry and Molecular Biology, Cumming School of Medicine, Faculty of Kinesiology, University of Calgary, Calgary, AB T2N 4N1, Canada

**Keywords:** metabolomics, pregnancy, mental health, depression, anxiety, nuclear magnetic resonance, inductively coupled plasma-mass spectrometry, metabolism

## Abstract

Approximately 25% of individuals report poor mental health during their pregnancy or postpartum period, which may impact fetal neurodevelopment, birth outcomes, and maternal behaviors. In the present study, maternal serum samples were collected from pregnancies at 28–32 weeks gestation from the All Our Families (Alberta, Canada) cohort and assessed using nuclear magnetic resonance spectroscopy (^1^H-NMR) and inductively coupled plasma-mass spectrometry (ICP-MS). Individuals with poor mental health at 34–36 weeks gestation were age-matched with mentally healthy pregnant controls. Metabolites were examined against validated self-reported mental health questionnaires for associations with depressive symptoms (Edinburgh Perinatal Depression Scale) and anxiety symptoms (Spielberger State-Trait Anxiety Inventory). ^1^H-NMR metabolites were identified for depression (alanine, leucine, valine, methionine, phenylalanine, glucose, lactate, 3-hydroxybutyrate, and pyruvate) and anxiety (3-hydroxybutyrate). For ICP-MS, antimony and zinc were significant for depression and anxiety, respectively. Upon false discovery rate (FDR) correction at 10%, five ^1^H-NMR metabolites (alanine, leucine, lactate, glucose, and phenylalanine) for depression remained significantly increased. Although results warrant further validation, the identified metabolites may serve as a predictive tool for assessing mental health during pregnancy as earlier identification has the potential to aid intervention and management of poor mental health symptomology, thus avoiding harmful consequences to both mother and offspring.

## 1. Introduction

It is often assumed that pregnancy elicits happiness and joy for expectant mothers. However, for many expecting mothers, significant changes in physiology, anatomy, interpersonal relationships, and at times autonomy may lead to unanticipated challenges and difficulties [1]. While some individuals easily adapt to these changes, others may be increasingly susceptible to the onset or relapse of mental health disorders [2]. It is estimated that 25% of individuals report having poor mental health at some point between conception and 1 year postpartum [3].

The concern for mental health during pregnancy has attracted more attention in recent years [4]. Particularly, awareness of mental health in pregnancy has shifted from postpartum depression to a wide spectrum encapsulating depression, anxiety, and stress. Recent evidence shows that poor mental health throughout a pregnancy may influence fetal development and infant birth outcomes, such as birth weight and gestational age [5]. For instance, individuals who score high for depression or anxiety have an increased risk of spontaneous preterm birth compared to their healthy counterparts [6,7,8]. For at-term births, there is a two-fold increase in small for gestational age births among individuals who suffer from depression [9]. Poor mental health during pregnancy is also known to affect a range of postnatal outcomes including reduced breastfeeding, mother–child interactions, sleep and bonding [10,11]. Collectively, poor mental health creates a less-than-ideal environment for mothers and can adversely impact child development.

Diagnosing depression and anxiety is challenging under normal physiological states. However, this difficulty is often amplified in pregnancy where symptoms related to poor mental health can be confused with conventional pregnancy symptoms, such as loss of energy, changes in appetite, sleep disturbances, and fatigue [12,13]. Across both clinical and research domains, degrees of poor mental health can be assessed and diagnosed via clinical judgement, observation and self-reported questionnaires [14]. However, the extensive range of available assessment tools can introduce variability and inconsistency in the diagnosis and prediction of poor mental health [15].

On the other hand, as a novel approach, metabolomics can potentially be used to determine unique mental health-related conditions due to its high sensitivity and specificity [16,17,18]. A number of studies have recently emerged examining metabolomics in relation to poor mental health, with the anticipation that potential biomarkers for mental health evaluation can be uncovered [16,19,20]. In the present study, we examined whether maternal serum metabolite signatures could distinguish between healthy controls and individuals with symptoms of depression and anxiety. To our knowledge, this is the first such investigation into mental health parameters and metabolites in late pregnancy.

## 2. Materials and Methods

The All Our Families (AOF) study was approved by the Child Health Research Office and the Conjoint Health Research Ethics Board at the University of Calgary, and written informed consent was obtained from all participants. Eligibility was based on the individuals being adults (≥18 years of age), being less than 25 weeks of gestation at the time of recruitment, receiving prenatal care in Alberta, Canada and being willing to complete written questionnaires in English [21]. Written questionnaires were completed by participants between 34–36 weeks of gestation. From the questionnaires, mental health was assessed using two validated scales, the Edinburgh Perinatal Depression Scale (EPDS) [22] and the Spielberger State-Trait Anxiety Inventory (STAI), which will be further discussed in Section 2.1 [23].

Serum samples, collected from 28–32 weeks of gestation, were evaluated by nuclear magnetic resonance spectroscopy (^1^H-NMR) and inductively coupled plasma-mass spectrometry (ICP-MS). Blood was collected from the antecubital vein and serum was isolated by standard procedures. Samples were centrifuged and stored as serum at −80 °C until data analysis [24]. A total of 466 maternal serum samples were evaluated using ^1^H-NMR spectroscopy and 386 were evaluated using ICP-MS in a single-blinded manner. Following sample measurement, mental health codes classified based on EPDS and STAI scores from 34–36 weeks of gestation were retrospectively matched to the assessed samples.

To evaluate the two mental health indices of interest, a total of 275 participants were analyzed using a 2:1 ratio of healthy controls to poor mental health participants for ^1^H-NMR. For ICP-MS, 219 participants were analyzed using a 2:1 ratio of healthy controls to poor mental health participants. Although there was an overlap between ^1^H-NMR and ICP-MS data, some participants used in the ^1^H-NMR dataset were excluded from the ICP-MS dataset due to poor signal quality.

### 2.1. Mental Health Screening

Mental health was evaluated by two questionnaires: the EPDS and STAI. EPDS is a screening tool to detect major depression among pregnant and postpartum individuals [25,26]. The EPDS evaluates behaviors related to low energy, guilt, anhedonia, sleep disturbances, and suicidal ideation. The range of possible scores for EPDS varies from a minimum score of 0 to a maximum score of 30, with higher scores indicating greater severity of depression [22]. Likewise, the STAI is a reliable and valid screening tool to detect anxiety among pregnant and postpartum individuals [26]. The STAI measures the degree to which individuals feel unease, worry, and tension. The range of possible scores for STAI varies from a minimum score of 20 to a maximum score of 80, with higher scores reflecting greater severity of anxiety symptoms [27]. Cutoffs relating to all mental health outcomes were predetermined by known clinical entities as previously published. For EPDS, individuals were stratified into two categories: unlikely to have depression (<13, *n* = 65), and probable depression (≥13, *n* = 34) [28,29]. For STAI, individuals were stratified into two categories: unlikely to have anxiety (<40, *n* = 173) and probable anxiety (≥40, *n* = 96) [23]. As perceived stress is known to accompany both depression and anxiety, Cohen’s Perceived Stress Inventory (PSS) score was included as a covariate [30].

### 2.2. Proton Nuclear Magnetic Resonance Spectroscopy (^1^H-NMR)

Samples were prepared as previously described [31,32]. Briefly, serum samples were placed in centrifugal filtering devices (NanoSep Omega 3K Membrane, Pall Biotech, Mississauga, ON, Canada). Starting volume for all serum samples was 200 μL. Upon centrifugation (Eppendorf 5415D, Hamberg, Germany) of the samples for 60 min at 10,000 rpm, 100 μL of D_2_O was added to the filtered sample and centrifuged for 45 min. 130 μL of 0.5 M Sample Buffer (NaH_2_PO_4_·H_2_O and 3-(trimethylsilyl)-1-propane sulfonic acid sodium salt, NaDSS) was added to the filtrate for frequency locking and to maintain a neutral pH of 7.4 and 10 μL of 1 M sodium azide (NaN_3_) was added to the filtrate as an antimicrobial agent. Filtrates were then brought up to 600 μL by D_2_O. 1D ^1^H spectra were recorded on a Bruker 600 MHz Avance II spectrometer (600.22 MHz, 5 mm TXI probe, 298 K. Billerica, MA, USA) equipped with a NMR case sample changer, inverse triple-resonance probe and z-gradients. Spectra were pre-processed using Chenomx NMR Suite Version 9.02 (Chenomx Inc., Edmonton, AB, Canada) by setting the DSS peak at 0 ppm and verifying that the peak is symmetrical and less than 2 Hz broad as previously described [32,33]. Upon baseline correction and automated phasing, metabolite assignment and quantification were conducted with a previously assigned serum spectrum and the 600 MHz in-house Chenomx library database [34].

### 2.3. Inductively Coupled Plasma-Mass Spectrometry (ICP-MS)

Inductively coupled plasma-mass spectrometry (ICP-MS) was used to profile ions found in serum as previously described [35]. Briefly, 12.5 μL of serum was transferred to 4.9 mL 2% nitric acid solution and vortexed. Samples were centrifuged for 10 min at 10,000 rpm. Ion analysis was performed using an ICP-MS, PlasmaQuant MS Elite Spectrometer (Analytik, Jena, Germany) using Seronorm trace element serum (Sero, Billingstad, Norway) as a quality control. A Seronorm standard curve was performed to find relative counts of the micronutrients, and metal and trace ions. A 2% nitric acid solution (blank) and a positive control were run every 5 samples. Samples were analyzed in the normal and integrated Collision Reaction Cell (iCRC) mode to attenuate most of the polyatomic interferences. All elements were subtracted by the nearest blank (2% nitric acid). As signals from the ICP-MS lose counts/second over time, all samples were multiplied by the ratio of their nearest control to the average of all the controls for the specific run. Any element with a coefficient of variation of less than 30% was included in the analysis.

### 2.4. Statistical Analysis

Statistical analysis of ^1^H-NMR data was carried out in R version 4.1.2 (The R Foundation for Statistical Computing, Vienna, Austria), SIMCA 17 (Umetrics, Umea, Sweden), SPSS (V28.0.1.) and GraphPad Prism 9.3.1. (San Diego, CA, USA). Multivariate data analysis, in the form of principal component analysis, was applied to identify potential outliers and strong group differences among mental health groups (control against depression or anxiety). All data were normalized by sum and logarithmically transformed. For ^1^H-NMR data, any metabolites in which over 50% of the data had missing values were excluded. For the remaining metabolites with missing values, data were imputed by *k*-nearest neighbors’ algorithm [36]. Differences in metabolites between two independent groups of mental health status were assessed by *t*-tests. Statistical significance was established at *p* < 0.05. The resulting *p*-values were corrected for multiple testing using false discovery rate (FDR) correction at a 10% level. MetaboAnalyst 5.0 was used to perform a pathway enrichment analysis to better interpret the meaning of the altered metabolites. Multivariate logistic modelling was also used to analyze significant results with either the EPDS or the STAI groups with also adjusting for various maternal characteristics (e.g., pre-pregnancy BMI, pre-pregnancy smoking status, maternal age, household income, mid-pregnancy perceived stress, and mid-pregnancy depression or anxiety). Statistical analysis was repeated for ICP-MS data. Descriptive statistics were used to describe maternal health and birth outcomes. Continuous data are reported as mean ± SD. Categorical data are reported as a percentage.

## 3. Results

### 3.1. Discriminating Metabolites Associated with Depression

To evaluate the impact of depression on serum profiles, individuals were stratified into two categories based on their EPDS score: those unlikely to have depression (<13) and those with probable depression (≥13). An EPDS cut-off of 13 has been shown to have high specificity in previous studies examining pregnant individuals [28]. In sum, 34 individuals were found to have probable depression and were then matched with 65 individuals who were unlikely to have depression as comparators. Table 1 displays participant characteristics stratified by EPDS groups. Individuals with probable depression were reported to have significantly higher mid-pregnancy anxiety and perceived stress scores, a greater likelihood of exceeding gestational weight gain guidelines, shorter gestational periods, and an increased likelihood of preterm births when compared to their unlikely to be depressed counterparts.

**Figure 1:**
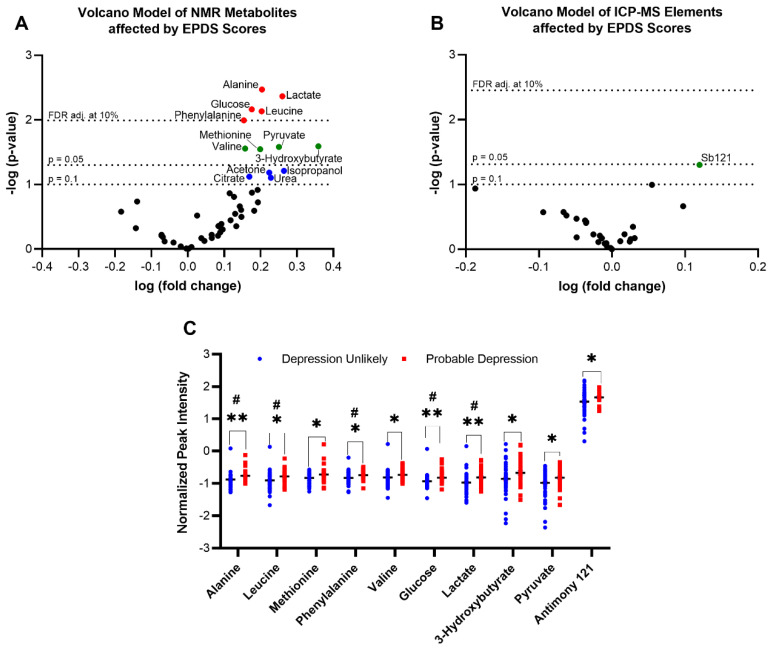
Association of Edinburgh Perinatal Depression Scale (EPDS) scores to serum metabolites. Significant metabolites selected by volcano plot with log fold change (*x*-axis) and −log(*p*-value) (*y*-axis); from ^1^H-NMR data (*p* < 0.05) (**A**) and from ICP-MS data (**B**). Metabolites remaining significant upon FDR (0.10) correction are also shown. Significantly differentially expressed metabolites and the direction of change as determined by parametric *t*-tests between depression unlikely and probable depression groups are shown in (**C**). * *p* < 0.05, ** *p* < 0.01 from *t*-tests; # FDR corrected significance (0.10).

To identify significant metabolite changes in the preliminary dataset, volcano plots displaying signal (e.g., log-fold-change) against noise-adjusted/standardized signal (e.g., t-statistic or −log_10_(*p*-value) from univariate *t*-tests) were created. This allowed for visual documentation of the most meaningful fold changes that are also significant amongst groups. Of detected ^1^H-NMR serum metabolites, alanine, leucine, methionine, phenylalanine, valine, glucose, lactate, 3-hydroxybutyrate, and pyruvate were found to be significantly increased in individuals with probable depression (Figure 1A). Although acetone, isopropanol, citrate, and urea did not meet statistical significance, they were also tentatively explored due to their close proximity to *p <* 0.1 and their interaction with covariates. For ICP-MS, the only significant metalloid was antimony (Sb121) (Figure 1B). Furthermore, all unadjusted *p*-values from the *t*-tests were corrected for multiple testing using an FDR correction at a 10% level [37]. Alanine, leucine, lactate, glucose, and phenylalanine retained significance upon FDR correction. Results of depression unlikely and probable depression groups for both ^1^H-NMR and ICP-MS data are shown in Figure 1C.

**Figure 2:**
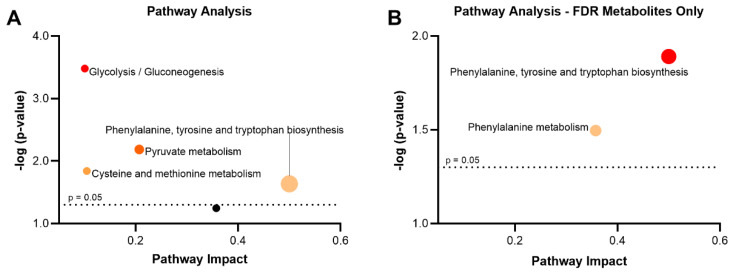
Metabolic pathway analysis of differential metabolites in third-trimester serum perturbed by unlikely and probable depression groups; from all significantly altered metabolites (**A**) and from significant metabolites upon FDR correction (**B**). Significant pathways (*p* < 0.05, pathway impact > 0.1) are labelled and denoted by varying colors and sizes to represent significance and impact, respectively.

Upon identifying several potential biomarkers, a pathway enrichment analysis (MetaboAnalyst 5.0) was generated to better interpret the meaning of the altered metabolites [38]. Results highlighted four significantly altered pathways (*p* < 0.05, pathway impact > 0.1) to be involved between unlikely and probable depression groups (Figure 2A). Within these pathways, phenylalanine, tyrosine and tryptophan biosynthesis (*p* = 0.023, impact 0.500), pyruvate metabolism (*p* = 0.007, impact 0.207), cysteine and methionine metabolism (*p* = 0.014, impact 0.105) and glycolysis/gluconeogenesis (*p* = 3.297 × 10^−4^, impact 0.101) were prominent and potentially indicate metabolic perturbations present with depression. The five metabolites which remained significant upon FDR correction were involved in two metabolic pathways (*p* < 0.05, pathway impact > 0.1): phenylalanine, tyrosine and tryptophan biosynthesis (*p* = 0.013, impact 0.500) and phenylalanine metabolism (*p* = 0.032, impact 0.357) (Figure 2B).

Although the present study was largely preliminary in nature, the influence of participant covariates on metabolite outcomes was also of interest. To this end, a multivariate modelling approach was employed wherein the resultant crude model was adjusted for covariates. Six covariates were selected based on their availability and clinical importance to depression based on previous literature [39,40,41,42,43,44]. Covariates included pre-pregnancy body mass index (BMI, kg/m^2^), pre-pregnancy smoking status, maternal age, household income, mid-pregnancy perceived stress scores, and mid-pregnancy anxiety scores. As shown in Table 2, it was found that alanine, methionine, glucose, and pyruvate remained significant upon all covariate adjustments when using unadjusted *p*-values. In addition, urea and citrate with unadjusted *p* < 0.1 became significant once adjusted for all covariates. Upon FDR correction at 10%, denoted as *q*-values, only pyruvate remained significant upon all covariate adjustment.

### 3.2. Discriminating Metabolites Associated with Anxiety

To investigate the extent that anxiety would alter the serum metabolome, individuals were stratified into two categories based on their STAI score: those unlikely to have anxiety (<40) and those with probable anxiety (≥40) in a 2:1 age-matched ratio. A total of 96 individuals with probable anxiety were age-matched with 173 individuals unlikely to have anxiety. Table 3 displays participant characteristics stratified by STAI groups. Individuals with probable anxiety were reported to have significantly higher mid-pregnancy depression and stress scores, greater pre-pregnancy BMIs, smaller household incomes, and an increased number of preterm births compared to their mentally healthy counterparts.

**Figure 3:**
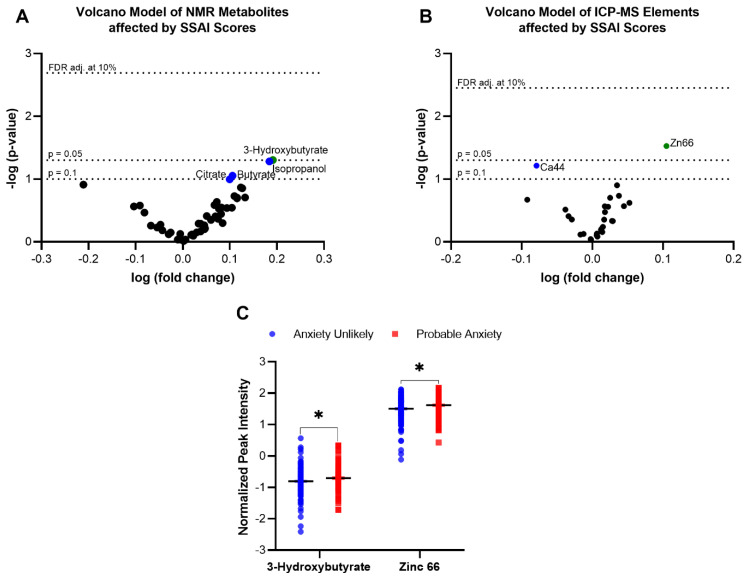
Association of Spielberger State-Trait Anxiety Inventory (STAI) scores to serum metabolites. Significant metabolites selected by volcano plot with log fold change (*x*-axis) and −log(*p*-value) (*y*-axis); from ^1^H-NMR data (**A**) and from ICP-MS data (**B**). Significantly expressed metabolites were determined by parametric *t*-tests between anxiety unlikely and probable anxiety groups (**C**). * *p* < 0.05.

To gain insight into differential metabolites between anxiety unlikely and probable anxiety groups, volcano models were constructed with the ^1^H-NMR and ICP-MS data (Figure 3A,B). Of the detected serum metabolites, only 3-Hydroxybutyrate and zinc were found to be significant (Figure 3C). As their raw *p*-values less than or equal to 0.10, isopropanol, butyrate, and citrate were also investigated with multivariate modelling with covariate adjustment. Upon FDR correction at 10%, no results remained significant.

Anxiety *p*-values were adjusted by six covariates as previously described for depression. From Table 4, it was found that only zinc 66 remained significant upon all covariate adjustments when using unadjusted *p*-values. Using *q*-values, no results were found to be significant.

## 4. Discussion

In the present study, we investigated serum metabolomic and metallomic patterns in relation to self-reported mental health questionnaires during late pregnancy, a period of greater susceptibility to depression and anxiety [2,3]. Although results yielded significant associations between serum metabolomics profiles for depression and anxiety, depression yielded the strongest metabolomic signature. Specifically, depression, as assessed by the self-reported EPDS, showed positive associations with alanine, glucose, lactate, leucine, methionine, phenylalanine, pyruvate, valine, 3-hydroxybutyrate, and antimony. After employing a conservative, multiple-testing approach, alanine, leucine, lactate, glucose, and phenylalanine remained statistically significant.

A potential hypothesis as to why these metabolites were different between the groups may relate to altered metabolism and energetic demands associated with depression. Corroborating this, an increasing number of studies show depression to be associated with mitochondrial dysfunction, oxidative stress, and inflammation [45,46,47]. All significant metabolites contribute to glycolysis and the tricarboxylic acid (TCA) cycle. Glucose is a principal energy source, while alanine may be shuttled to pyruvate. Likewise, phenylalanine and leucine may be converted to acetyl-CoA, and lactate is the end product of anaerobic glycolysis. This potential upregulation of glycolysis and the TCA cycle, as observed by the significantly elevated metabolites, may be due to the increased demand for energy in depression [48]. Another metabolite fitting with this hypothesis was the observed increase in 3-hydroxybutyrate with likely depression. 3-hydroxybutyrate is an efficient mitochondrial fuel that provides an alternate source of energy for the brain during times of energy deficit. It also participates in various signaling and regulatory roles through its actions on histone deacetylation and gene regulation [49]. Although significance was lost upon FDR correction, elevations in this metabolite are supported by a number of reports of 3-hydroxybutyrate being elevated in both adolescents and adults with depression [50,51,52]. Elevations in 3-hydroxybutyrate likely reflect extra energy demands, as well as insulin resistance that commonly accompanies depression [53].

Alterations in energy utilization, particularly an increase in serum leucine and valine, have been observed in metabolomic assessments of inflammation [54,55]. For decades, inflammation has been proposed as a potential contributor to the pathophysiology of depression as inflammatory cytokines, released by stress-induced stimuli, disrupt serotonin, dopamine, and glutamate pathways in the brain [56]. Specifically, it is posited that abnormal energy utilization may promote inflammatory processes [57], elevate cortisol levels via the hypothalamus–pituitary–adrenal axis [58,59], and reduce thyroid hormone levels [60], all of which increase the likelihood of depression. As the present study was able to detect profiles associated with elevated EPDS scores, metabolomics may also be of value in predicting when individuals who are likely to experience depression may relapse in the future. Notably, Mocking and colleagues [61] show that a metabolomic signature could predict those patients most likely to relapse up to two-and-a-half years in the future with greater than 90% accuracy. This finding is of interest to pregnancy, as individuals who have prenatal depression are much more likely to experience postpartum depression [62]. Therefore, metabolomics profiling may be of particular value in identifying depression in those individuals who may require continued monitoring following birth.

As depression is influenced by several biological, psychological, and social factors, our study also sought to determine the relationship of metabolites with common covariates. For example, pre-pregnancy obesity is associated with an increased risk of experiencing prenatal depression (odds ratio 1.33) [63]. Six potential covariates (pre-pregnancy BMI, pre-pregnancy smoking, household income, maternal age, and mid-pregnancy stress and anxiety) were controlled in logistic models. Alanine, methionine, pyruvate, and glucose remained significant upon fully controlling for the effects of all six covariates prior to FDR adjustment. This finding indicates that these biomarkers may provide additional discriminatory information, and thus a closer relationship with prenatal depression and greater EPDS scores [64,65], not seen for the other metabolites which would benefit from further investigation.

Like depression, pregnancy is also a time of greater susceptibility to anxiety. Currently, there is a limited number of studies investigating alterations to the metabolome associated with complex mood and anxiety disorders among pregnant individuals [66]. Although lost upon multiple-testing correction, anxiety, as evaluated by the self-reported STAI, initially showed positive associations for 3-hydroxybutyrate and zinc. Other investigated metabolites of interest (*p* < 0.1) included calcium, isopropanol, butyrate and citrate. The smaller number of significant associations observed between maternal serum and prenatal anxiety likely indicates small metabolite effect sizes. However, given the preliminary nature of this work, we have opted to briefly discuss both results pertaining to prenatal anxiety.

Anxiety was associated with an increase in 3-hydroxybutyrate. As with depression, this metabolite appears to be significantly reflective of psychosomatic stressors, such as fatigue, headaches, and palpitations, that is often caused by mood and anxiety disorders [51]. It is also possible that this signal is due to depression rather than anxiety per se. Indeed, there is considerable overlap with anxious individuals experiencing more depression and stress in comparison to their non-anxious counterparts. In support of this, the *p*-value for 3-hydroxybutyrate (*p* = 0.049) was minimally altered with corrections for body mass index, smoking and income (range; *p* = 0.054–0.084), but completely lost when adjusted for stress and depression (*p* = 0.272). 

A micronutrient altered with anxiety was zinc. Zinc was found to be positively associated with STAI, even after all covariate corrections. Zinc is an essential micronutrient that regulates the central nervous system and neuronal functions [67]. There is growing evidence that zinc deficiency can lead to symptomology related to poor mental health, with zinc supplementation providing anti-anxiety-like effects [68,69,70,71]. However, to the best of our knowledge, this zinc link is still inconclusive in pregnant populations as observed in Roomruangwong and colleagues [72], whose study identified no correlation (*p* = 0.729) between zinc levels and anxiety scores in the prenatal and postpartum periods. In addition, most pregnant individuals in the present study (~87%, data not shown) were on prenatal vitamin supplements, all of which contain zinc in some form. Given this, elevated serum zinc levels in pregnancy with anxiety could reflect lower absorption or prenatal transfer to the fetus. Serum elevations in zinc in the present study may also coincide with the lower calcium (Ca) observed in pregnancy with anxiety (*p* = 0.031 with BMI adjustment). Zinc and Ca have been suggested to compete for the same absorption sites [73] and the intestinal absorption of Ca is known to be lower with zinc supplementation [74]. Regardless of the potential mechanisms, the linkages between zinc, Ca, and anxiety require further elucidation in pregnancy.

## 5. Conclusions

In this preliminary study, we demonstrate distinct metabolomic and metallomic signatures based on questionnaire responses for depression and anxiety observed from serum analyzed by ^1^H-NMR spectroscopy and ICP-MS. We also provided some insights into metabolite-mental health relationships. With the utility of metabolomics becoming more prominent in clinical settings, this study contributes to our current understanding that poor mental health can have on our physiology. Although further validation should occur, the implicated metabolites could eventually serve as biomarkers in identifying individuals at risk for poor mental health during their pregnancy.

## Figures and Tables

**Table 1:** AOF Population Characteristics stratified by Depression.

	Depression Unlikely ^1^ (*n* = 65)	Probable Depression ^1^ (*n* = 34)	*p*-Value
Maternal Characteristics			
Mid-pregnancy anxiety, *n* ≥ 40 STAI ^2^ Score (%)	12 (19)	31 (91)	<0.001 *
Mid-pregnancy stress, *n* ≥ 14 PSS ^3^ Score (%)	27 (42)	34 (100)	<0.001 *
Pre-pregnancy BMI, mean (SD), kg/m^2^	24.3 (5.15)	27.4 (7.42)	0.05
Met GWG Guidelines ^4^, *n* yes (%)	46 (71)	16 (48)	0.02 *
Household income, *n* ≥ $80,000 (%)	43 (68)	21 (66)	0.75
Education, *n* only high school completion (%)	8 (12)	6 (18)	0.27
Maternal age at delivery, mean (SD), years	31.6 (5.51)	31.3 (5.91)	0.83
Pre-pregnancy smoking, *n* yes (%)	14 (22)	5 (15)	0.41
Birth Characteristics			
Sex, *n* male (%)	29 (45)	20 (59)	0.18
Gestational age at birth, mean (SD), weeks	38.8 (1.51)	37.8 (2.46)	0.03 *
Birth weight, mean (SD), grams	3316 (490)	3098 (717)	0.13
Large for gestational age ^5^, *n* LGA (%)	4 (7.1)	3 (9.1)	0.71
Small for gestational age ^6^, *n* SGA (%)	4 (7.1)	4 (12)	0.46
Preterm birth (≤36 weeks), *n* preterm (%)	4 (6.2)	8 (24)	0.02 *

^1^ Edinburgh Perinatal Depression Scale classified by unlikely to have depression score (<13, *n* = 65) and probable depression (≥13, *n* = 34). ^2^ Spielberger State-Trait Anxiety Inventory Score (STAI) is used to assess anxiety. ^3^ Cohen’s Perceived Stress Inventory Score (PSS). ^4^ Meeting gestational weight gain (GWG) guidelines were based on the Institute of Medicine guidelines and pre-pregnancy BMI. ^5^ LGA: birthweight above the 90th percentile of sex-specific birth weight. ^6^ SGA: birthweight below the 10th percentile of sex-specific birth weight. BMI, body mass index. * *p* < 0.05.

**Table 2:** Multivariate Logistic Modelling of Relevant EPDS Metabolites.

Results with *p* < 0.1	Unadj. *p*-Value	FDR *q*-Value	BMI ^1^		Smoking ^2^		Maternal Age		Household Income ^3^		Stress ^4^ + Anxiety ^5^		All	
			*p*-Value	*q*-Value	*p*-Value	*q*-Value	*p*-Value	*q*-Value	*P*-Value	*q*-Value	*p*-Value	*q*-Value	*p*-Value	*q*-Value
Alanine	**0.003**	**0.090**	**0.002**	**0.088**	**0.003**	**0.082**	**0.003**	**0.082**	**0.001**	**0.049**	**0.045**	0.315	**0.012**	0.245
Leucine	**0.007**	**0.090**	**0.016**	0.112	**0.013**	0.114	**0.012**	0.118	**0.015**	0.123	0.094	0.512	0.156	0.640
Methionine	**0.028**	0.155	**0.004**	**0.088**	**0.014**	0.114	**0.009**	0.110	**0.009**	**0.088**	**0.036**	0.315	**0.042**	0.294
Phenylalanine	**0.010**	**0.099**	**0.009**	**0.088**	**0.010**	0.114	**0.015**	0.123	**0.007**	**0.086**	0.188	0.709	0.196	0.640
Valine	**0.028**	0.155	**0.028**	0.172	**0.037**	0.201	**0.039**	0.191	**0.026**	0.182	0.293	0.763	0.222	0.640
Glucose	**0.007**	**0.090**	**0.008**	**0.088**	**0.005**	**0.082**	**0.005**	**0.082**	**0.007**	**0.086**	**0.025**	0.315	**0.018**	0.245
Lactate	**0.004**	**0.090**	**0.006**	**0.088**	**0.004**	**0.082**	**0.005**	**0.082**	**0.003**	**0.074**	0.129	0.588	0.066	0.404
3-BHB ^6^	**0.026**	0.155	**0.049**	0.240	**0.030**	0.184	**0.026**	0.182	**0.040**	0.192	0.139	0.588	0.222	0.640
Pyruvate	**0.026**	0.155	0.067	0.278	**0.030**	0.184	**0.039**	0.191	**0.031**	0.190	**0.010**	0.315	**0.002**	**0.098**
Antimony 121	0.052	0.877	0.055	0.935	0.109	0.972	0.114	0.965	0.075	0.946	0.223	0.659	0.204	0.640
Acetone	0.066	0.292	0.068	0.278	0.061	0.270	0.058	0.258	**0.050**	0.204	0.372	0.829	0.374	0.746
IPA ^7^	0.061	0.292	0.168	0.492	0.066	0.270	0.077	0.314	0.126	0.386	0.817	0.960	0.690	0.867
Citrate	0.076	0.297	**0.016**	0.112	**0.043**	0.211	**0.038**	0.191	**0.041**	0.192	**0.040**	0.315	**0.037**	0.294
Urea	0.079	0.297	0.087	0.328	0.112	0.392	0.110	0.415	0.094	0.327	**0.042**	0.315	**0.037**	0.294

^1^ Pre-pregnancy body mass index (BMI, kg/m^2^). ^2^ Pre-pregnancy smoking is defined as smoking up to a year before conception date. ^3^ Household income is defined as total income before taxes being less than $39,999, $40,000–79,999, or over $80,000. ^4^ Cohen’s Perceived Stress Inventory Score (PSS). ^5^ Spielberger State-Trait Anxiety Inventory score is used to assess anxiety. ^6^ BHB denotes hydroxybutyrate. ^7^ IPA denotes isopropanol. Bold denotes *p* < 0.05 or *q* < 0.1.

**Table 3:** AOF Population Characteristics stratified by Anxiety.

	Anxiety Unlikely ^1^ (*n* = 173)	Probable Anxiety ^1^ (*n* = 96)	*p*-Value
Maternal Characteristics			
Mid-pregnancy depression, *n* ≥ 13 EPDS ^2^ Score (%)	2 (1.2)	31 (33)	<0.001 *
Mid-pregnancy stress, *n* ≥ 14 PSS ^3^ Score (%)	60 (35)	88 (92)	<0.001 *
Pre-pregnancy BMI, mean (SD), kg/m^2^	24.3 (5.20)	25.6 (5.71)	0.037 *
Met GWG Guidelines ^4^, *n* yes (%)	105 (62)	58 (61)	0.14
Household income, *n* ≥ $80,000 (%)	126 (76)	56 (60)	0.017 *
Education, *n* only high school completion (%)	17 (.9.8)	15 (16)	0.23
Maternal age at delivery, mean (SD), years	31.7 (4.30)	31.8 (4.96)	0.83
Pre-pregnancy smoking, *n* yes (%)	24 (14)	21 (22)	0.096
Birth Characteristics			
Sex, *n* male (%)	91 (53)	48 (50)	0.68
Gestational age at birth, mean (SD), weeks	38.9 (1.90)	38.5 (2.04)	0.11
Birth weight, mean (SD), grams	3307 (544)	3257 (599)	0.52
Large for gestational age ^5^, *n* LGA (%)	12 (7.8)	9 (10)	0.53
Small for gestational age ^6^, *n* SGA (%)	16 (10)	9 (10)	0.95
Preterm birth (≤36 weeks), *n* preterm (%)	10 (5.9)	13 (14)	0.033 *

^1^ Spielberger State-Trait Anxiety Inventory classified by unlikely to have anxiety score (<40, *n* = 173) and probable anxiety (≥40, *n* = 96). ^2^ Perinatal Depression Scale (EPDS) is used to assess depression. ^3^ Cohen’s Perceived Stress Inventory Score (PSS). ^4^ Meeting gestational weight gain guidelines were based on the Institute of Medicine guidelines and pre-pregnancy body mass index (BMI, kg/m^2^), ^5^ LGA: birthweight above the 90th percentile of sex-specific birth weight. ^6^ SGA: birthweight below the 10th percentile of sex-specific birth weight. * *p* < 0.05.

**Table 4:** Multivariate Logistic Modelling of Relevant STAI Metabolites.

Results with *p* < 0.1	Unadj. *p*-Value	FDR *q*-Value	BMI ^1^		Smoking ^2^		Maternal Age		Household Income ^3^		Stress ^4^ + Depression ^5^		All	
			*p*-Value	*q*-Value	*p*-Value	*q*-Value	*p*-Value	*q*-Value	*p*-Value	*q*-Value	*p*-Value	*q*-Value	*p*-Value	*q*-Value
3-BHB ^6^	**0.049**	0.765	**0.054**	0.822	0.075	0.787	0.056	0.756	0.084	0.732	0.272	0.956	0.493	0.952
Zinc 66	**0.030**	0.769	**0.049**	0.654	**0.036**	0.644	**0.030**	0.658	0.061	0.758	**0.050**	0.467	**0.036**	0.815
IPA ^7^	0.052	0.765	0.061	0.822	0.068	0.787	0.062	0.756	0.120	0.732	0.314	0.956	0.734	0.952
Butyrate	0.088	0.765	0.114	0.822	0.096	0.787	0.098	0.756	0.085	0.732	0.288	0.956	0.405	0.952
Citrate	0.100	0.765	0.129	0.822	0.126	0.787	0.124	0.756	0.153	0.732	0.548	0.956	0.758	0.952
Calcium 44	0.061	0.769	**0.031**	0.654	**0.046**	0.644	**0.047**	0.658	0.070	0.758	**0.033**	0.467	0.080	0.815

^1^ Pre-pregnancy body mass index (BMI, kg/m^2^). ^2^ Pre-pregnancy smoking is defined as up to a year before conception date. ^3^ Household income is defined as total income before taxes being less than $39,999, $40,000–79,999, or over $80,000. ^4^ Cohen’s Perceived Stress Inventory Score (PSS). ^5^ Edinburgh Perinatal Depression Scale (EPDS) is used to assess depression. ^6^ BHB denotes hydroxybutyrate. ^7^ IPA denotes isopropanol. Bold denotes *p* < 0.05 or *q* < 0.1.

## Data Availability

The data presented in this study are available on request. The All Our Families data are not publicly available due to privacy.
